# Kaempferol Attenuates Myocardial Ischemia–Reperfusion Injury by Suppressing Ferroptosis via the KEAP1–Nrf2–GPX4 Axis

**DOI:** 10.1002/kjm2.70234

**Published:** 2026-05-22

**Authors:** Yan‐Bo Zhao, Ling‐Ling Sun, Xiao‐Hua Shen, Min Wang, Mei‐Hui Wang, Lu‐Lu Liu, Kai Zhang

**Affiliations:** ^1^ Department of Cardiology, Sir Run Run Shaw Hospital Zhejiang University School of Medicine Hangzhou Zhejiang Province China; ^2^ Zhejiang Key Laboratory of Cardiovascular Intervention and Precision Medicine Hangzhou Zhejiang Province China; ^3^ Engineering Research Center for Cardiovascular Innovative Devices of Zhejiang Province Hangzhou Zhejiang Province China

**Keywords:** cardioprotection, ferroptosis, kaempferol, KEAP1–Nrf2–GPX4 axis, myocardial ischemia/reperfusion injury

## Abstract

Myocardial ischemia/reperfusion (I/R) injury is characterized by cardiomyocyte death, excessive oxidative stress, inflammation, and ferroptosis, which collectively limit the efficacy of reperfusion therapy. In this study, we investigated whether kaempferol (KAE), a natural flavonol with antioxidative and antiinflammatory properties, could protect against myocardial I/R injury by modulating the KEAP1–Nrf2–GPX4 signaling pathway. Using hypoxia/reoxygenation (H/R)‐injured AC16 cardiomyocytes and a mouse myocardial I/R model, we observed that KAE significantly enhanced cardiomyocyte viability, promoted Nrf2 nuclear translocation, and upregulated downstream antioxidant and antiferroptotic proteins, including HO‐1, NQO1, GPX4, and SLC7A11, while reducing KEAP1 and ACSL4 expression. KAE also markedly attenuated lipid peroxidation, iron accumulation, reactive oxygen species generation, apoptosis, and inflammatory responses in vitro and in vivo. These protective effects were partially abolished by Nrf2 knockdown and were replicated by the ferroptosis inhibitor ferrostatin‐1 (Fer‐1), indicating a ferroptosis‐dependent mechanism. Consistently, KAE pretreatment attenuated myocardial injury, preserved cardiac histology, restored GPX4 and SLC7A11 expression, and lowered serum cTnI, CK‐MB, TNF‐α, and IL‐6 levels in I/R mice. Taken together, these results demonstrate that KAE alleviates myocardial I/R injury by activating the KEAP1–Nrf2–GPX4 axis and suppressing ferroptosis and oxidative stress, highlighting its potential as a therapeutic agent in ischemic heart disease.

## 1 Introduction

Acute myocardial infarction (AMI), a leading cardiovascular disorder with high morbidity and a risk of sudden death, affects approximately 3.8% of individuals under 60 years and 9.5% of those over 60 worldwide [1, 2]. Although timely interventions such as thrombolysis, percutaneous coronary intervention, and coronary artery bypass grafting can restore coronary perfusion and improve outcomes, reperfusion itself can paradoxically cause additional cardiomyocyte injury, exacerbating cardiac dysfunction [3]. This pathological process, known as myocardial ischemia/reperfusion (I/R) injury, is characterized by arrhythmias, impaired contractility, intense inflammatory responses, and cardiomyocyte necrosis or apoptosis [4]. Consequently, there is an urgent need for novel therapeutic approaches that mitigate myocardial I/R injury and complement existing reperfusion strategies.

Ferroptosis, a regulated form of cell death distinct from apoptosis and necrosis, has emerged as a critical contributor to myocardial I/R injury [5, 6]. It is driven by iron‐dependent lipid peroxidation resulting from excessive oxidative stress and impaired antioxidant defenses [7]. During reperfusion, the sudden restoration of oxygen induces a ROS burst, which, together with disrupted iron homeostasis, triggers lipid peroxidation and compromises membrane integrity, ultimately leading to cardiomyocyte ferroptosis [8]. Accumulating evidence indicates that ferroptosis contributes substantially to myocardial injury, contractile dysfunction, and inflammatory amplification during I/R [9]. The KEAP1–Nrf2 pathway serves as a central defense against oxidative stress and ferroptosis [10]. Under basal conditions, Nrf2 is retained in the cytoplasm by KEAP1 and targeted for degradation [11]. In response to oxidative stress, Nrf2 dissociates from KEAP1 and translocates into the nucleus, where it transcriptionally activates a suite of cytoprotective genes, including GPX4 and SLC7A11 [12, 13]. SLC7A11, a key component of the cystine/glutamate antiporter (system Xc^−^), mediates cystine uptake for glutathione (GSH) synthesis, whereas GPX4 utilizes GSH to reduce lipid peroxides and prevent ferroptotic cell death, thereby maintaining redox homeostasis [14]. Dysregulation of this axis amplifies I/R‐induced oxidative damage and ferroptosis, whereas Nrf2 activation confers cardioprotection [15, 16, 17]. Accordingly, targeting the KEAP1–Nrf2–GPX4 axis to inhibit ferroptosis represents a promising therapeutic strategy for myocardial I/R injury.

Kaempferol (KAE) is a natural flavonol primarily derived from the rhizome of *Kaempferia galanga* L., and is also abundant in dietary sources such as broccoli, beans, and tomatoes [18, 19]. KAE has demonstrated antioxidative, antiinflammatory, and cardioprotective effects. In myocardial I/R models, it reduces oxidative stress and apoptosis, acting through mechanisms such as GSK‐3β inhibition [20], MAPK pathway modulation [21], and miR‐21‐mediated activation of the Notch/PTEN/Akt signaling axis [22]. Although these studies underscore its protective effects against conventional I/R injury pathways, the role of KAE in modulating ferroptosis in cardiomyocytes remains largely unexplored. In noncardiac contexts, KAE has been shown to suppress ferroptosis by upregulating SLC7A11 and GPX4, thereby mitigating neuronal [23] and endothelial [24] injury, as well as liver damage via Nrf2 activation [25]. These findings suggest a potential role of KAE in regulating ferroptosis, but whether such effects occur in cardiomyocytes during myocardial I/R injury, and whether the KEAP1–Nrf2–GPX4 axis is involved, remain to be determined.

The present study aims to fill this critical gap by investigating whether KAE modulates myocardial ferroptosis during I/R injury through the KEAP1–Nrf2–GPX4 axis. We will also explore how the effects of KAE intersect with oxidative stress and inflammation. This study represents a novel mechanistic insight that may support the development of KAE as a therapeutic agent for myocardial I/R injury.

## 2 Materials and Methods

### 2.1 Cell Culture and KAE Treatment

AC16 human cardiomyocytes (ATCC) were cultured in Dulbecco's Modified Eagle's Medium (DMEM, Gibco) supplemented with 10% fetal bovine serum (FBS, Gibco) and 1% penicillin–streptomycin. Cells were maintained at 37°C in a humidified incubator with 5% CO_2_ and passaged every 2–3 days. AC16 cells, although immortalized, retain key cardiac characteristics and are commonly used to study in vitro myocardial I/R and oxidative stress [26, 27]. Experiments were performed when cells reached 70%–80% confluence. KAE (purity ≥ 98%, Yuanye Bio‐Technology Co. Ltd., Shanghai, China) was dissolved in DMSO to prepare a 10 mM stock solution and stored at −20°C. For preliminary experiments, cells were treated with KAE at 0, 1, 5, 10, 20, or 40 μM for 6, 12, 24, or 48 h.

### 2.2 Cell Viability Assay

Cell viability was evaluated using the Cell Counting Kit‐8 (CCK‐8; Dojindo, Tokyo, Japan). AC16 cells were plated in 96‐well plates at a density of 5 × 10^3^ cells/well and treated with KAE as described. After treatment, 10 μL of CCK‐8 reagent was added to each well, followed by incubation for 2 h at 37°C. Absorbance at 450 nm was measured with a microplate reader, and cell viability was calculated as follows: Cell viability (%) = [(OD sample − OD blank)/(OD control − OD blank)] × 100%.

### 2.3 Cell Transfection and Treatment

For mechanistic studies, AC16 cells were transfected with Nrf2‐targeting siRNA (si‐Nrf2) or scrambled control (si‐NC) using Lipofectamine 3000 (Invitrogen) following the manufacturer's instructions. Transfection was performed 24 h prior to KAE pretreatment, and knockdown efficiency was confirmed by Western blot. Ferrostatin‐1 (Fer‐1, Selleck, Cat# S7243) was applied at 1 μM, 1 h before hypoxia/reoxygenation (H/R) in designated groups as a positive control for ferroptosis inhibition.

### 2.4 H/R Injury Model

To mimic myocardial I/R injury in vitro, AC16 cells were subjected to 16 h of hypoxia (1% O_2_, 5% CO_2_, 94% N_2_, 37°C) followed by 6 h of reoxygenation under normoxic conditions (21% O_2_, 5% CO_2_, 37°C). Cells in KAE‐pretreated groups received either low‐dose or high‐dose KAE for 24 h before H/R. Ferrostatin‐1 and si‐Nrf2 interventions were applied as previously described.

### 2.5 Immunofluorescence Assay

AC16 cardiomyocytes were fixed with 4% paraformaldehyde for 15 min at room temperature and blocked with 1% BSA for 1 h. Cells were then incubated overnight at 4°C with anti‐Nrf2 (1:50, 1639‐1‐AP, Proteintech, USA). After three PBS washes, cells were incubated with a fluorescent secondary antibody for 1–2 h at room temperature in the dark, followed by nuclear staining with DAPI (Life Technologies). Fluorescence images were captured using a fluorescence microscope, and Nrf2 nuclear translocation was quantified by the ratio of nuclear to cytoplasmic fluorescence intensity.

### 2.6 Flow Cytometric Analysis

Following the indicated treatments, AC16 cardiomyocytes were harvested by trypsinization, washed twice with cold PBS, and subjected to flow cytometric analysis to evaluate lipid peroxidation, intracellular ROS production, and apoptosis. Lipid peroxidation was assessed using the C11‐BODIPY 581/591 fluorescent probe. Cells were incubated with the probe at 37°C for 30 min in the dark, washed with PBS, and analyzed by flow cytometry. Oxidation‐induced shifts in fluorescence emission from red to green were quantified, and lipid peroxidation levels were expressed as mean fluorescence intensity (MFI) relative to controls. Intracellular ROS production was measured using the DCFH‐DA probe. Following incubation at 37°C for 30 min in the dark and removal of excess dye, fluorescence signals were detected by flow cytometry. ROS levels were quantified as MFI relative to the control group. Apoptosis was evaluated using an Annexin V‐FITC/propidium iodide (PI) apoptosis detection kit. Cells were resuspended in binding buffer and incubated with Annexin V‐FITC and PI for 15 min at room temperature in the dark. Total apoptotic cells (early + late) were calculated as percentages of the total cell population.

### 2.7 Biochemical Analysis of Ferroptosis‐Related Indicators

After treatments, AC16 cells were lysed on ice using cell lysis buffer. Supernatants were collected after centrifugation at 12,000*g* for 10 min at 4°C. Intracellular Fe^2+^ levels were measured using a Ferrous Iron Colorimetric Assay Kit (Abcam, ab83366) and normalized to total protein. Lipid peroxidation was evaluated by MDA levels (Beyotime, S0131S). GSH and GSSG levels were measured (Beyotime, S0053) to calculate the GSH/GSSG ratio, reflecting intracellular redox status.

### 2.8 Animal Experiments

Male C57BL/6J mice (8–10 weeks old, 22–25 g) were obtained from an accredited animal facility and housed under standard conditions with free access to food and water. Only male mice were used in this study to avoid the potential influence of sex hormones, such as estrogen, on myocardial I/R injury and related signaling pathways. All procedures were approved by the Institutional Animal Care and Use Committee of Sir Run Run Shaw Hospital, Zhejiang University School of Medicine (Approval No. SRRSH20250725031, Zhejiang, China) and performed in accordance with the Guide for the Care and Use of Laboratory Animals. Mice were randomly assigned into four groups (*n* = 8 per group): Sham, I/R, I/R + low‐dose KAE (I/R + L‐KAE), and I/R + high‐dose KAE (I/R + H‐KAE). Myocardial I/R injury was induced as previously described [28]. Briefly, under general anesthesia and mechanical ventilation, a left thoracotomy was performed to expose the heart, and the left anterior descending (LAD) coronary artery was ligated with a 7–0 silk suture for 30 min to induce ischemia, followed by 24 h of reperfusion. Sham mice underwent the same procedure without LAD occlusion. For pharmacological intervention, KAE was dissolved in DMSO and diluted with saline (final DMSO < 0.1%, v/v). Mice in the treatment groups received a single intraperitoneal injection of KAE (50 mg/kg for the low‐dose group and 100 mg/kg for the high‐dose group) 1 h before reperfusion. These doses were selected based on previous studies showing that flavonoids, including KAE, are safe and exert cardioprotective effects in rodent models [29]. Sham and I/R groups received an equivalent volume of vehicle (0.1% DMSO in saline) following the same schedule. At the end of reperfusion, mice were euthanized under deep anesthesia. Blood was collected by cardiac puncture, centrifuged at 3000*g* for 15 min at 4°C to obtain serum, and stored at −80°C for subsequent biochemical and ELISA measurements. The hearts were rapidly excised, rinsed in cold PBS, and sectioned. Portions of the left ventricle were fixed in 4% paraformaldehyde for histological analysis, while others were snap‐frozen in liquid nitrogen for protein extraction, ELISA, and biochemical assays.

### 2.9 ELISA Assays

Serum levels of myocardial injury markers and inflammatory cytokines were quantified using commercial ELISA kits according to the manufacturers' instructions. For myocardial injury assessment, mouse cardiac troponin I (cTnI) and creatine kinase‐MB (CK‐MB) levels were measured using ELISA kits obtained from Biomatik (cat. no. EKU11622, Canada) and G‐Biosciences (cat. no. IT5598, USA), respectively. For inflammatory cytokine analysis, TNF‐α and IL‐6 levels were determined. In vitro, culture supernatants from AC16 cells were collected, centrifuged at 1000*g* for 10 min, and analyzed using human TNF‐α (R&D Systems, cat. no. DTA00C) and IL‐6 (R&D Systems, cat. no. D6050) ELISA kits. In vivo, mouse serum samples were collected by centrifugation at 3000*g* for 15 min at 4°C and analyzed using mouse TNF‐α (R&D Systems, cat. no. DY410) and IL‐6 (R&D Systems, cat. no. M6000B) ELISA kits.

### 2.10 Hematoxylin and Eosin (H&E) Staining

Myocardial tissue samples were fixed in 4% paraformaldehyde for 24 h at room temperature, dehydrated through graded ethanol, cleared in xylene, and embedded in paraffin. Paraffin‐embedded hearts were sectioned at 4 μm thickness, mounted on slides, and deparaffinized in xylene followed by rehydration through a graded ethanol series to distilled water. Sections were stained with hematoxylin for 5 min, rinsed in running tap water, differentiated briefly in 1% acid alcohol, blued in 0.2% ammonia water, and counterstained with eosin for 2 min. After dehydration and clearing, slides were coverslipped and examined under a light microscope (Olympus BX53). Myocardial histopathological changes, including tissue damage, necrosis, and inflammatory infiltration, were evaluated in a blinded manner. Representative images were captured from at least five randomly selected fields per section.

### 2.11 TUNEL Assay

Apoptotic cardiomyocytes in myocardial tissue were detected using a TUNEL assay kit (KeyGen Biotech, cat. no. KGA702) following the manufacturer's instructions. Briefly, paraffin‐embedded heart sections (4 μm) were deparaffinized, rehydrated, and incubated with TUNEL reaction mixture. Nuclei were counterstained with DAPI, and TUNEL‐positive cells were quantified in at least five randomly selected fields per section under a fluorescence microscope.

### 2.12 Immunohistochemical Staining

Paraffin‐embedded myocardial sections (4 μm) were deparaffinized, rehydrated, and subjected to antigen retrieval using citrate buffer (pH = 6.0) at 95°C for 20 min. Sections were blocked with 5% bovine serum albumin for 30 min and incubated overnight at 4°C with primary antibodies against KEAP1 (Abcam, cat. no. ab66620, 1:200), Nrf2 (Cell Signaling Technology, cat. no. 12721, 1:200), or GPX4 (Abcam, cat. no. ab125066, 1:200). After washing, sections were incubated with HRP‐conjugated secondary antibodies for 1 h at room temperature and visualized using DAB substrate. Nuclei were counterstained with hematoxylin. For quantification, five randomly selected fields per section were captured under a light microscope at ×200 magnification. KEAP1 and GPX4 were quantified as mean optical density (MOD, IOD/area) using ImageJ software, whereas Nrf2 was quantified as the percentage of positive cells per field.

### 2.13 Western Blot Analysis

Total proteins were extracted from AC16 cardiomyocytes or mouse myocardial tissues using RIPA lysis buffer supplemented with protease and phosphatase inhibitors (Beyotime, China). For analysis of Nrf2 subcellular distribution, nuclear and cytoplasmic proteins were separately isolated using a commercial Nuclear and Cytoplasmic Protein Extraction Kit (Beyotime, China) according to the manufacturer's instructions. Protein concentrations were determined by the BCA assay (Thermo Fisher Scientific, USA). Equal amounts of protein (20–40 μg) were resolved by SDS‐PAGE and transferred onto PVDF membranes (Millipore, USA). Membranes were blocked with 5% nonfat milk in TBST for 1 h at room temperature and incubated overnight at 4°C with primary antibodies against KEAP1 (1:1000, Abcam, ab139729), Nrf2 (1:1000, Cell Signaling Technology, #12721), HO‐1 (1:1000, Abcam, ab13248), NQO1 (1:1000, Abcam, ab80588), GPX4 (1:1000, Proteintech, 67763‐1‐Ig), ACSL4 (1:1000, Abcam, ab155282), SLC7A11 (1:1000, Proteintech, 26864‐1‐AP), cleaved caspase‐3, or NLRP3 (1:1000, ab263899, Abcam). After washing, membranes were incubated with HRP‐conjugated secondary antibodies (1:5000, Abcam) for 1 h at room temperature. Protein bands were visualized using an enhanced chemiluminescence detection system (ECL, Thermo Fisher Scientific) and imaged with a ChemiDoc imaging system (Bio‐Rad, USA). Band intensities were quantified using ImageJ software (NIH, USA) and normalized to GAPDH for total and cytoplasmic proteins or Lamin B1 for nuclear proteins.

### 2.14 Statistical Analysis

All data are presented as mean ± standard deviation (SD). Statistical analyses were performed using GraphPad Prism 8.0 (GraphPad Software, San Diego, CA, USA). Comparisons between two groups were conducted using unpaired two‐tailed Student's *t*‐test, while multiple group comparisons were performed using one‐way ANOVA followed by Tukey's post hoc test. A value of *p* < 0.05 was considered statistically significant. All experiments were repeated at least three times independently, and investigators were blinded to group allocation during data collection and analysis.

## 3 Results

### 3.1 Effects of KAE on AC16 Cardiomyocyte Viability

**FIGURE 1:**
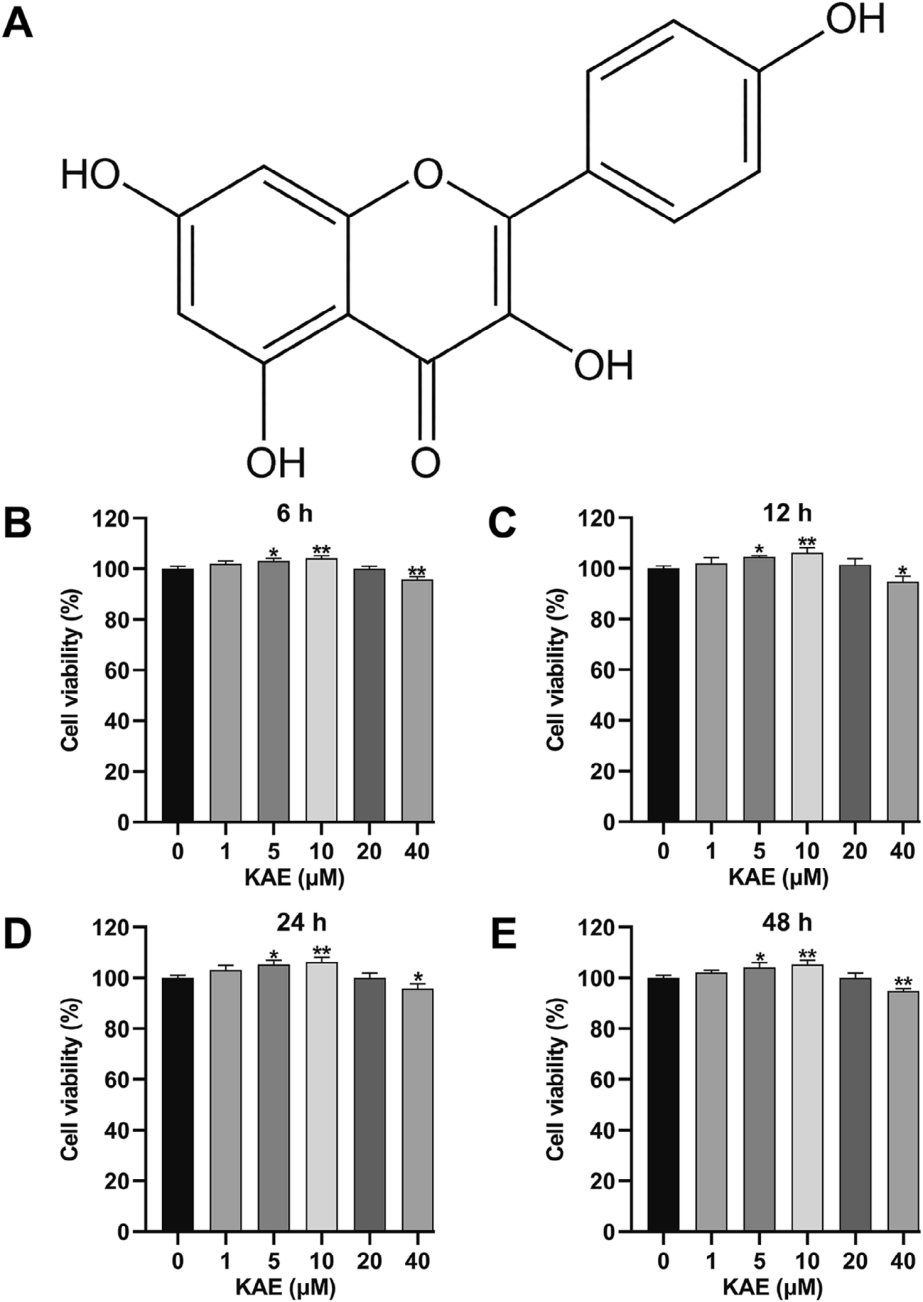
Effects of KAE on AC16 cardiomyocyte viability. (A) Chemical structure of KAE. (B–E) AC16 cells were treated with KAE at 0, 1, 5, 10, 20, and 40 μM for 6, 12, 24, and 48 h, respectively, and cell viability was assessed using the CCK‐8 assay. Data are presented as mean ± SD (*n* = 3 independent experiments). *p < 0.05, **p < 0.01, vs. control. Low‐dose (L‐KAE, 5 μM) and high‐dose (H‐KAE, 10 μM) concentrations were selected for subsequent experiments based on these results.

The chemical structure of KAE is shown in Figure 1A. To evaluate its effects on cardiomyocyte viability, AC16 cells were treated with KAE at concentrations of 0, 1, 5, 10, 20, and 40 μM for 6, 12, 24, and 48 h, and cell viability was assessed using the CCK‐8 assay (Figure 1B–E). KAE at 5 μM and 10 μM modestly increased cell viability across all time points (*p* < 0.05), while 20 μM had no significant effect. The highest concentration, 40 μM, slightly reduced viability. Based on these results, 5 and 10 μM were chosen as the low‐dose (L‐KAE) and high‐dose (H‐KAE) for subsequent experiments, respectively.

### 3.2 KAE Activates the KEAP1–Nrf2 Antioxidant Signaling Pathway in Cardiomyocytes Under H/R Conditions

**FIGURE 2:**
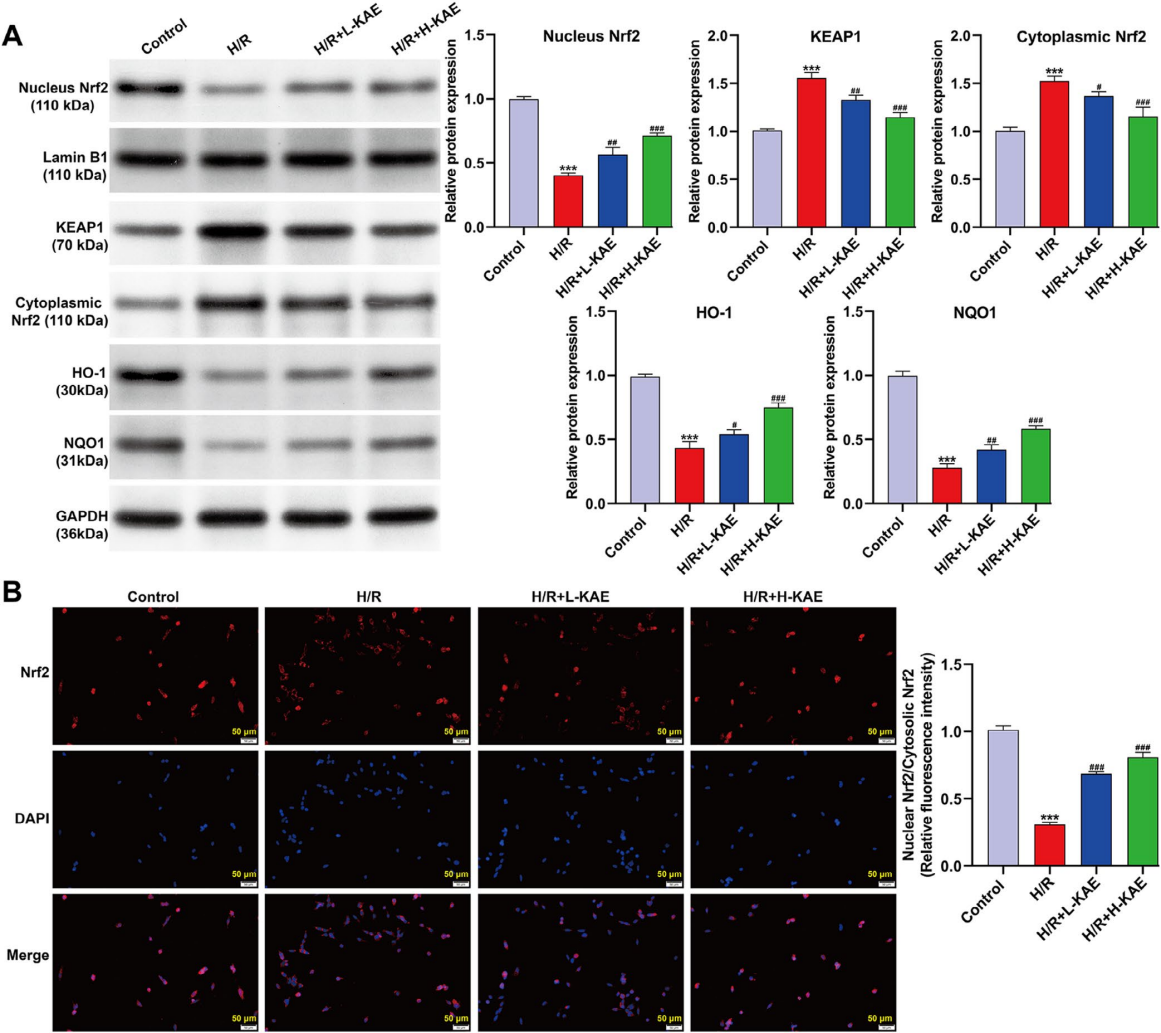
KAE activates the KEAP1–Nrf2 antioxidant signaling pathway in cardiomyocytes subjected to H/R. Cells were pretreated with KAE at low (5 μM, L‐KAE) or high (10 μM, H‐KAE) concentrations for 24 h prior to hypoxia/reoxygenation (H/R) exposure. (A) Representative Western blot images and quantitative analysis of KEAP1, cytoplasmic Nrf2 (Cyto‐Nrf2), nuclear Nrf2 (Nuc‐Nrf2), HO‐1, and NQO‐1 in AC16 cardiomyocytes. Protein expression was normalized to GAPDH (cytoplasmic proteins) or Lamin B1 (nuclear proteins). (B) Immunofluorescence staining of Nrf2 (red) with nuclei counterstained by DAPI (blue). Data are presented as mean ± SD (*n* = 3 independent experiments). ***p < 0.001, vs. control; #*p* < 0.05, ##*p* < 0.01, ###*p* < 0.001, vs. H/R.

To investigate whether KAE exerts cardioprotective effects through modulation of the KEAP1–Nrf2 signaling pathway during H/R injury, AC16 cardiomyocytes were pretreated with L‐KAE or H‐KAE prior to H/R exposure. The protein expression levels of KEAP1, cytoplasmic and nuclear Nrf2, as well as downstream antioxidant enzymes HO‐1 and NQO‐1, were examined by Western blot analysis (Figure 2A). As expected, H/R stimulation markedly increased KEAP1 expression and significantly suppressed Nrf2 nuclear accumulation compared with the control group, accompanied by reduced expression of HO‐1 and NQO‐1. KAE pretreatment dose‐dependently reversed these effects. Specifically, both L‐KAE and H‐KAE significantly downregulated KEAP1 protein levels while markedly enhancing Nrf2 nuclear translocation. Consistently, the expression of the Nrf2 downstream targets HO‐1 and NQO‐1 was significantly upregulated in KAE‐treated cells, with the H‐KAE group exhibiting a more pronounced effect. To further confirm the effect of KAE on Nrf2 subcellular localization, immunofluorescence staining was performed (Figure 2B). In control cells, Nrf2 was predominantly localized in the cytoplasm, whereas H/R exposure markedly impaired Nrf2 nuclear translocation. KAE pretreatment significantly promoted Nrf2 accumulation in the nucleus under H/R conditions, as evidenced by enhanced nuclear fluorescence intensity, further supporting the activation of the KEAP1–Nrf2 signaling axis.

### 3.3 KAE Suppresses H/R‐Induced Ferroptosis via the KEAP1–Nrf2–GPX4 Axis

**FIGURE 3:**
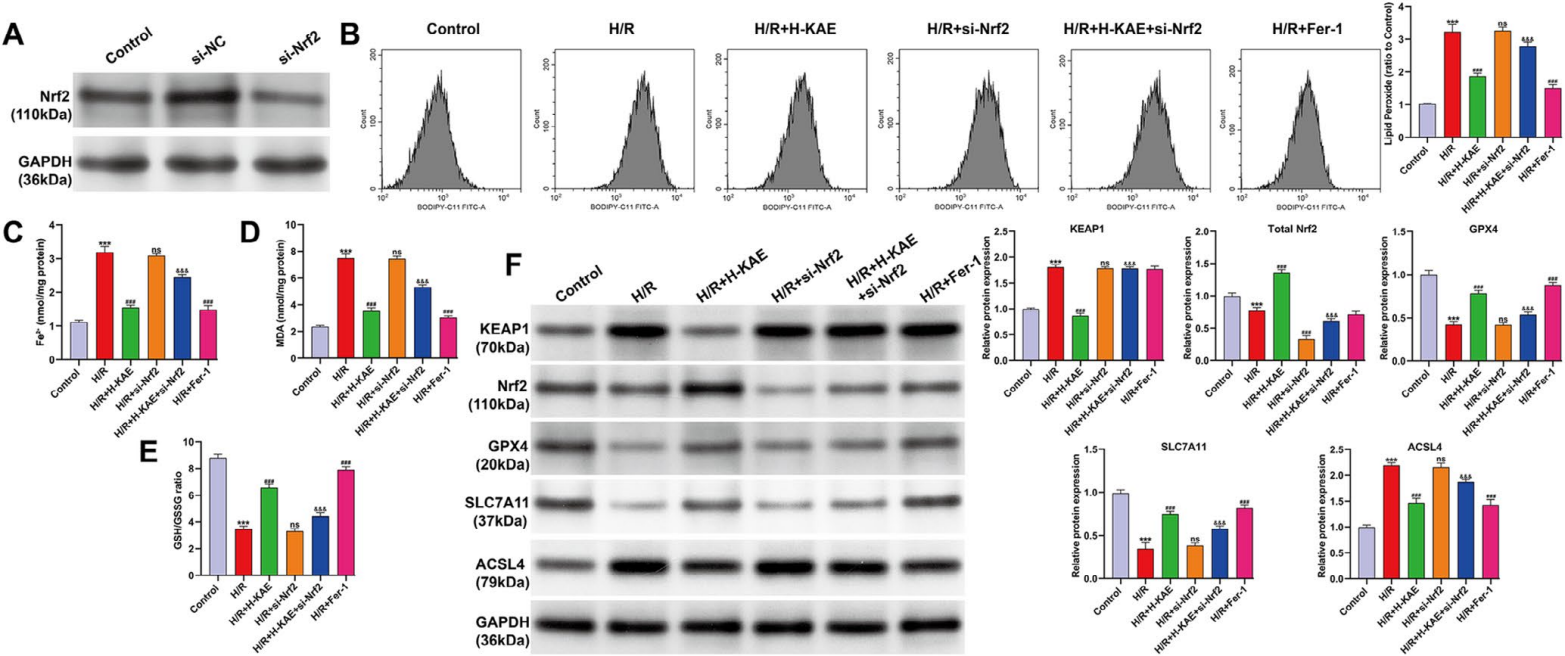
KAE suppresses H/R‐induced ferroptosis via the KEAP1–Nrf2–GPX4 axis. (A) Western blot analysis of total Nrf2 protein levels in Control, si‐NC, and si‐Nrf2 groups. (B–F) AC16 cells were pretreated with H‐KAE, transfected with si‐Nrf2, or treated with the ferroptosis inhibitor Ferrostatin‐1 (Fer‐1) prior to H/R, which were assigned to six experimental groups: Control, H/R, H/*R* + H‐KAE, H/*R* + si‐Nrf2, H/*R* + H‐KAE + si‐Nrf2, and H/*R* + Fer‐1. (B) Lipid peroxidation was assessed using C11‐BODIPY staining followed by flow cytometric analysis. (C) Intracellular Fe^2+^ content (nmol/mg protein) was measured as an indicator of iron accumulation. (D) Malondialdehyde (MDA) levels (nmol/mg protein) were determined as a marker of lipid peroxidation. (E) GSH/GSSG ratio was measured to evaluate cellular redox status. (F) Western blot analysis of GPX4, SLC7A11, ACSL4, KEAP1, and Nrf2. Protein expression was normalized to GAPDH. Data are presented as mean ± SD (*n* = 3 independent experiments). ***p < 0.001, vs. control; ###*p* < 0.001, vs. H/R; &&&*p* < 0.001, vs. H/*R* + H‐KAE; ns indicates no significant difference.

To examine whether the KEAP1–Nrf2 pathway mediates the regulation of KAE on ferroptosis under H/R conditions, we first confirmed efficient Nrf2 knockdown in AC16 cardiomyocytes using si‐Nrf2, which significantly reduced total Nrf2 protein compared with si‐NC and control cells (Figure 3A). Ferroptosis‐associated lipid peroxidation, assessed by C11‐BODIPY staining and flow cytometry, was markedly increased following H/R exposure, whereas H‐KAE pretreatment substantially attenuated this increase (Figure 3B). In parallel, intracellular Fe^2+^ content and MDA levels, both elevated by H/R, were significantly reduced by KAE (Figure 3C,D). Consistently, the GSH/GSSG ratio, indicative of cellular redox status, was decreased under H/R and restored by KAE treatment (Figure 3E). The ferroptosis inhibitor Fer‐1 produced comparable protective effects, confirming the involvement of ferroptotic mechanisms. Notably, Nrf2 silencing under H/R had minimal impact on these functional markers alone but markedly impaired the protective effects of KAE, resulting in elevated lipid peroxidation, iron accumulation, and oxidative stress compared with H‐KAE‐treated cells. Western blot analysis further demonstrated that H/R downregulated the ferroptosis‐suppressive proteins GPX4 and SLC7A11 while upregulating ACSL4 (Figure 3F). Pretreatment with H‐KAE reversed these changes, concomitantly reducing KEAP1 expression and restoring total Nrf2 levels. In contrast, Nrf2 knockdown largely abolished KAE‐mediated regulation of GPX4, ACSL4, and SLC7A11, whereas Fer‐1 restored these proteins independently of KEAP1–Nrf2 signaling. Collectively, these findings indicate that KAE mitigates H/R‐induced ferroptosis in AC16 cardiomyocytes primarily through activation of the KEAP1–Nrf2–GPX4 axis.

### 3.4 KAE Attenuates H/R‐Induced Oxidative Stress, Apoptosis, and Inflammatory Responses in AC16 Cardiomyocytes

**FIGURE 4:**
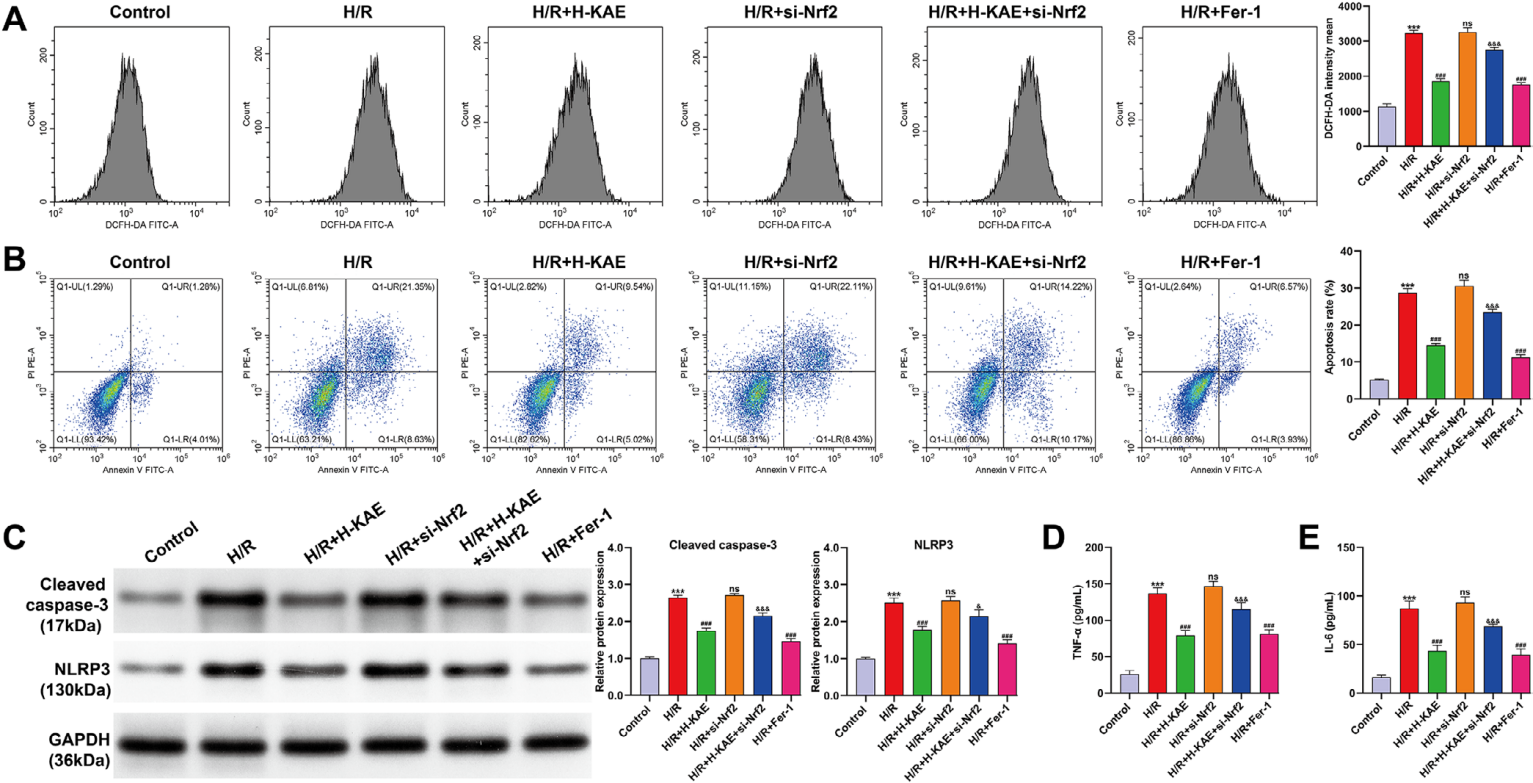
KAE attenuates H/R‐induced oxidative stress, apoptosis, and inflammatory responses in AC16 cardiomyocytes. AC16 cells were assigned to six experimental groups: Control, H/R, H/*R* + H‐KAE, H/*R* + si‐Nrf2, H/*R* + H‐KAE + si‐Nrf2, and H/*R* + Fer‐1. (A) Intracellular reactive oxygen species (ROS) levels were measured using DCFH‐DA staining and flow cytometry. (B) Apoptosis was assessed by flow cytometric analysis of Annexin V‐positive cells. (C) Western blot analysis of cleaved caspase‐3 and NLRP3. Protein expression was normalized to GAPDH. (D–E) ELISA analysis of TNF‐α (D) and IL‐6 (E) levels. Data are presented as mean ± SD (*n* = 3 independent experiments). ***p < 0.001, vs. control; ###*p* < 0.001, vs. H/R; &*p* < 0.05, &&&*p* < 0.001, vs. H/*R* + H‐KAE; ns indicates no significant difference.

Building on the finding that KAE suppresses H/R‐induced ferroptosis via the KEAP1–Nrf2–GPX4 axis, we next investigated its effects on oxidative stress, apoptosis, and inflammation. AC16 cells were exposed to H/R with or without H‐KAE pretreatment, Nrf2 knockdown, or Fer‐1 treatment. Intracellular ROS levels were measured using DCFH‐DA staining and flow cytometry (Figure 4A). H/R markedly increased ROS compared with normoxic controls, which were significantly reduced by KAE pretreatment. Nrf2 silencing partially attenuated this antioxidant effect, while Fer‐1 treatment produced a similar decrease, suggesting that ferroptosis contributes to ROS generation during H/R. Apoptosis was evaluated by flow cytometric analysis of Annexin V‐positive cells (Figure 4B) and by Western blot detection of cleaved caspase‐3 (Figure 4C). H/R increased apoptosis and caspase‐3 activation, both of which were suppressed by KAE. Nrf2 knockdown partially reversed the antiapoptotic effect, whereas Fer‐1 exhibited comparable protection. Inflammatory responses were examined by Western blot analysis of NLRP3 (Figure 4C) and ELISA measurement of TNF‐α (Figure 4D) and IL‐6 (Figure 4E). H/R elevated NLRP3 levels and cytokine release, which were markedly attenuated by KAE. Silencing Nrf2 partially diminished these antiinflammatory effects, whereas Fer‐1 also reduced NLRP3 and cytokine levels, indicating that ferroptosis contributes to inflammatory activation under H/R. Overall, these results demonstrate that KAE alleviates H/R‐induced oxidative stress, apoptosis, and inflammation in AC16 cardiomyocytes, predominantly through Nrf2‐dependent mechanisms.

### 3.5 KAE Alleviates Histological Injury and Apoptosis in Myocardial I/R Mice

**FIGURE 5:**
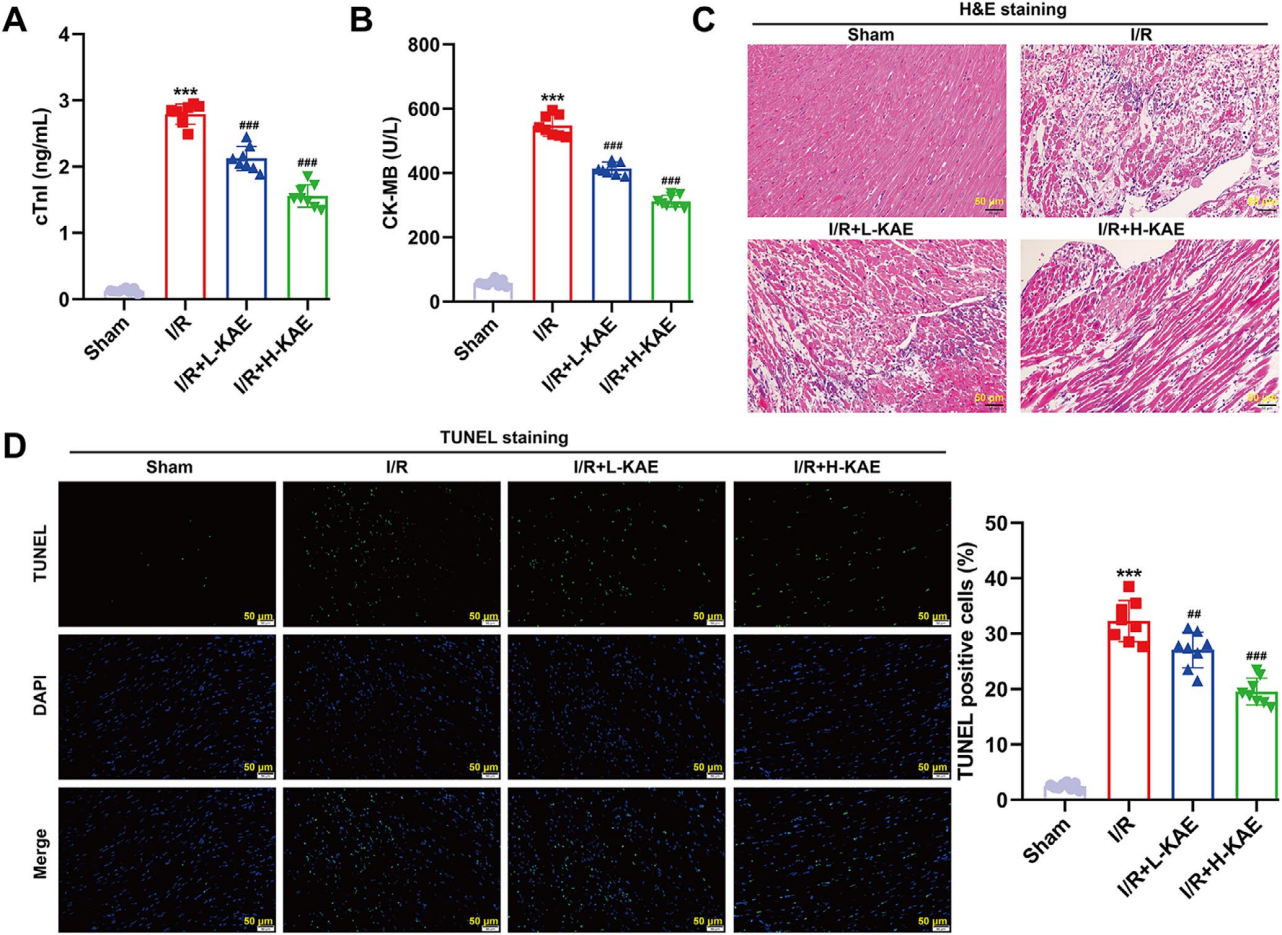
KAE alleviates myocardial injury and apoptosis in I/R mice. Male C57BL/6J mice were randomly assigned to four groups (*n* = 8 per group): Sham, I/R, I/*R* + low‐dose KAE (50 mg/kg, i.p.), and I/*R* + high‐dose KAE (100 mg/kg, i.p.). KAE was administered 1 h prior to reperfusion. Myocardial I/R injury was induced by ligation of the left anterior descending (LAD) coronary artery for 30 min followed by 24 h of reperfusion. (A–B) Serum levels of cardiac injury markers cTnI (A) and CK‐MB (B) were measured to assess myocardial damage. (C) H&E staining of myocardial tissue. (D) Cardiomyocyte apoptosis was assessed by TUNEL staining. Scale bar = 50 μm. Data are presented as mean ± SD (*n* = 8 independent experiments). ***p < 0.001, vs. Sham; ##*p* < 0.01, ###*p* < 0.001, vs. I/R.

To assess the in vivo cardioprotective effects of KAE, C57BL/6J mice were subjected to myocardial I/R injury with or without pretreatment with low‐ or high‐dose KAE, while Sham mice underwent the same surgical procedure without LAD ligation. Serum markers of cardiac injury were measured first. I/R markedly elevated cTnI and CK‐MB levels, whereas KAE administration dose‐dependently reduced both markers (Figure 5A,B), indicating mitigation of myocardial damage. Histological evaluation further confirmed these effects. H&E staining revealed myocardial disarray, cardiomyocyte swelling, and interstitial edema in I/R mice, whereas KAE‐treated hearts showed preserved tissue architecture and reduced structural disruption (Figure 5C). TUNEL staining demonstrated a substantial increase in apoptotic cardiomyocytes following I/R, which was significantly attenuated by both low‐ and high‐dose KAE, with the high‐dose group exhibiting a stronger effect (Figure 5D). These findings indicate that KAE effectively alleviates myocardial injury and apoptosis in vivo.

### 3.6 KAE Activates the KEAP1–Nrf2–GPX4 Axis and Mitigates Inflammation in Myocardial I/R Mice

**FIGURE 6:**
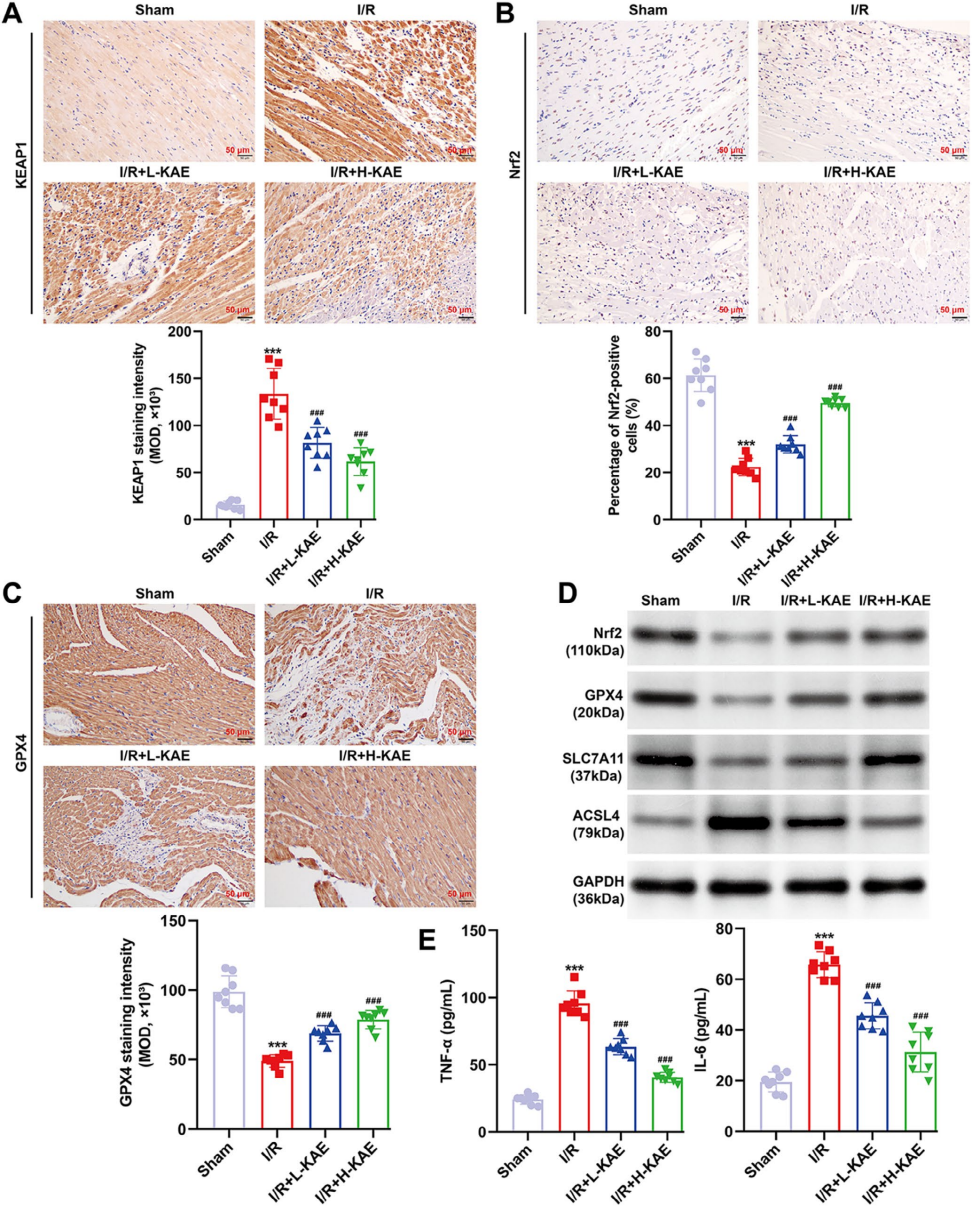
KAE activates the KEAP1–Nrf2–GPX4 axis and mitigates inflammation in myocardial I/R mice. Male C57BL/6J mice were randomly assigned to four groups (*n* = 8 per group): Sham, I/R, I/*R* + L‐KAE, and I/*R* + H‐KAE. (A–C) Immunohistochemical staining of KEAP1, Nrf2, and GPX4. KEAP1 and GPX4 were quantified as mean optical density (MOD), and Nrf2 as the percentage of positive cells. Scale bar = 50 μm. (D) Western blot analysis of Nrf2, GPX4, SLC7A11, and ACSL4. Protein expression was normalized to GAPDH. (E) ELISA analysis of TNF‐α and IL‐6. Data are presented as mean ± SD (*n* = 8 independent experiments). ***p < 0.001, vs. Sham; ###*p* < 0.001, vs. I/R.

To elucidate the in vivo mechanisms underlying the cardioprotective effects of KAE, myocardial tissue from Sham, I/R, I/R + low‐dose KAE, and I/R + high‐dose KAE mice was analyzed. Immunohistochemical staining was performed to assess the tissue distribution of KEAP1, Nrf2, and GPX4. KEAP1 expression was elevated in I/R myocardium, while Nrf2 nuclear localization was reduced; KAE treatment decreased KEAP1 and promoted Nrf2 accumulation in the nucleus in a dose‐dependent manner (Figure 6A,B). GPX4 staining demonstrated reduced expression in I/R myocardium, which was preserved by KAE administration (Figure 6C). Western blot analysis confirmed these observations, showing that I/R downregulated Nrf2, GPX4, and SLC7A11 while upregulating the proferroptotic enzyme ACSL4 (Figure 6D). KAE pretreatment restored Nrf2 and ferroptosis‐suppressive proteins and suppressed ACSL4 expression, consistent with activation of the KEAP1–Nrf2–GPX4 signaling axis. Inflammatory responses were evaluated by measuring serum TNF‐α and IL‐6 levels using ELISA assay. I/R markedly increased both cytokines, whereas KAE treatment dose‐dependently reduced their concentrations, indicating attenuation of systemic inflammation (Figure 6E). Taken together, these findings support that KAE activates the KEAP1–Nrf2–GPX4 pathway in vitro.

## 4 Discussion

Despite advances in reperfusion therapies, myocardial I/R injury remains a leading cause of morbidity and mortality worldwide [30]. The paradoxical tissue damage caused by reperfusion involves oxidative stress, inflammation, and cell death, limiting the overall efficacy of current treatments [31]. Natural compounds with multitarget effects, such as Kaempferol (KAE), have shown promise in mitigating these pathological processes by reducing oxidative stress, suppressing apoptosis, and modulating inflammatory responses [20, 21]. Our findings corroborate these protective effects, demonstrating that KAE preserves cardiomyocyte viability, attenuates apoptosis, and alleviates histological injury in both in vitro H/R and in vivo murine I/R models, supporting its potential as a cardioprotective therapeutic agent.

Ferroptosis, characterized by iron‐dependent lipid peroxidation, is increasingly recognized as a critical contributor to myocardial I/R injury [5, 6, 7, 8, 9]. Emerging studies indicate that KAE can inhibit ferroptosis through distinct molecular mechanisms in different contexts. For instance, Zhang et al. found that KAE suppressed ferroptosis in lung epithelial cells by inhibiting NSUN7‐mediated m5C methylation of TFRC, thereby limiting iron uptake and lipid peroxidation [32]. Similarly, Lou et al. demonstrated that KAE alleviates lipid metabolism disorders and ferroptotic cell death via activation of the AMPK–CD36 axis, highlighting its role in coordinating energy and lipid homeostasis [33]. In a cardiotoxicity model, Zhang et al. showed that KAE mitigates doxorubicin‐induced myocardial injury by reducing mitochondrial ROS‐dependent ferroptosis [34]. Consistent with these findings, our study demonstrates that KAE mitigates ferroptosis, as evidenced by reduced lipid peroxidation, iron accumulation, and restoration of GPX4 and SLC7A11 levels in H/R‐treated AC16 cells and I/R mouse hearts.

The KEAP1–Nrf2 pathway serves as a central regulator of cellular antioxidant defense and ferroptosis. Under oxidative stress, Nrf2 dissociates from KEAP1, translocates into the nucleus, and transcriptionally activates cytoprotective genes such as GPX4 and SLC7A11, which directly detoxify lipid peroxides and maintain redox balance [12, 16]. Pharmacological or genetic activation of Nrf2 has been shown to reduce myocardial I/R injury and suppress ferroptotic cell death in preclinical models [35, 36, 37]. Our findings indicate that KAE enhances Nrf2 nuclear accumulation and restores GPX4 and SLC7A11 expression, consistent with these studies. This dual modulation of upstream signaling (KEAP1–Nrf2) and downstream ferroptotic executors provides a mechanistic advantage over single‐target agents like Fer‐1, which directly inhibit lipid peroxidation but do not restore antioxidant signaling. Similar mechanisms have been reported as follows: Zhang et al. demonstrated that KAE attenuates oxygen–glucose deprivation/reoxygenation‐induced neuronal ferroptosis by activating the Nrf2/SLC7A11/GPX4 axis, augmenting antioxidant capacity and suppressing lipid peroxidation [23]. Furthermore, in a model of cigarette smoke extract‐induced lung injury, KAE was shown to modulate the Nrf2/NCOA4/GPx4 axis, thereby reducing the labile iron pool, restoring GPX4 activity, and inhibiting ferroptosis through Nrf2‐dependent transcriptional activation [25]. In hepatic models of drug‐induced injury, KAE activated Nrf2 signaling and upregulated GPX4 to suppress hepatocyte ferroptosis, providing protection against oxidative damage and iron overload [24]. These evidences support the notion that KAE‐mediated activation of the KEAP1–Nrf2–GPX4 axis is a conserved protective strategy. Notably, our study provides integrated in vitro and in vivo evidence that KAE simultaneously suppresses KEAP1, promotes Nrf2 nuclear translocation, and restores downstream ferroptosis effectors (GPX4, SLC7A11) in myocardial I/R injury. In contrast, other flavonoids, including curcumin [38], quercetin [39], and baicalein [40], primarily modulate individual components of the Nrf2 pathway, highlighting KAE's coordinated, multilevel regulation of the entire axis under ischemic stress.

Despite these encouraging findings, several limitations warrant consideration. First, although our study focused on the KEAP1–Nrf2–GPX4 axis and ferroptosis and used immortalized AC16 cells, whose physiology may not fully reflect primary cardiomyocytes, the protective effects of kaempferol were confirmed in vivo, supporting the physiological relevance of our findings and warranting future validation in primary or hiPSC‐derived cardiomyocytes. Second, the in vivo experiments addressed only acute I/R injury, without evaluation of long‐term outcomes such as cardiac remodeling or progression to heart failure. Third, cardiac functional parameters, such as echocardiographic measurements (e.g., ejection fraction and fractional shortening), were not assessed in this study, which may limit the evaluation of the overall cardioprotective efficacy of KAE in vivo. Fourth, the pharmacokinetics, tissue distribution, and bioavailability of KAE were not characterized, which may limit clinical translation. Finally, more comprehensive analyses of lipid metabolism and iron homeostasis are needed to fully elucidate the antiferroptotic mechanisms of KAE. Addressing these limitations in future studies will be essential for advancing KAE as a potential therapeutic agent for myocardial I/R injury.

In summary, our study demonstrates that KAE confers cardioprotection against myocardial I/R injury by activating the KEAP1–Nrf2–GPX4 signaling axis, thereby inhibiting ferroptosis and attenuating oxidative stress, apoptosis, and inflammatory responses. These findings provide mechanistic evidence supporting KAE as a promising therapeutic candidate for mitigating I/R‐induced myocardial damage. Further investigations incorporating additional signaling pathways and chronic I/R models are warranted to fully establish the translational potential of KAE in cardiovascular therapy.

## Funding

This research received funding from the Natural Science Foundation of Zhejiang Province (LMS26H020007).

## Ethics Statement

All procedures were approved by the Institutional Animal Care and Use Committee of Sir Run Run Shaw Hospital, Zhejiang University School of Medicine (approval no. SRRSH20250725031, Zhejiang, China) and conducted in accordance with the National Institutes of Health Guide for the Care and Use of Laboratory Animals.

## Conflicts of Interest

The authors declare no conflicts of interest.

## Data Availability

The data that support the findings of this study are available from the corresponding author upon reasonable request.
